# Acute kidney injury and point-of-care ultrasound in liver cirrhosis: redefining hepatorenal syndrome

**DOI:** 10.1093/ckj/sfae112

**Published:** 2024-04-15

**Authors:** Eduardo Josué Banegas-Deras, Jaime Mazón-Ruiz, Gregorio Romero-González, Juan Carlos Ruiz-Cobo, Clara Sanz-García, Mara Serrano-Soto, Emilio Sánchez, Eduardo R Argaiz

**Affiliations:** Nephrology Department, Carmen y Severo Ochoa Public Hospital, Cangas del Narcea, Spain; Nephrology Department, Central University Hospital of Asturias, Oviedo, Spain; Nephrology Department, Germans Trias i Pujol University Hospital, Badalona, Spain; International Renal Research Institute of Vicenza, Vicenza, Italy; Liver Unit, Vall d'Hebron University Hospital, Barcelona, Spain; Department of Medicine, Universitat Autònoma de Barcelona, Barcelona, Spain; Nephrology Department, Grande Covián de Arriondas Hospital, Arriondas, Spain; International Renal Research Institute of Vicenza, Vicenza, Italy; Nephrology Department, Marqués de Valdecilla University Hospital, Santander, Spain; Nephrology Department, Cabueñes University Hospital, Gijón, Spain; Tecnológico de Monterrey, Escuela de Medicina y Ciencias de la Salud, Mexico City, Mexico; Departamento de Nefrología y Metabolismo Mineral, Instituto Nacional de Ciencias Médicas y Nutrición Salvador Zubirán, Mexico City, Mexico

**Keywords:** acute kidney injury, cirrhosis, hepatorenal syndrome, point-of-care ultrasound, venous excess ultrasound score

## Abstract

Acute kidney injury (AKI) in patients with cirrhosis is a diagnostic challenge due to multiple and sometimes overlapping possible etiologies. Many times, diagnosis cannot be made based on case history, physical examination or laboratory data, especially when the nephrologist is faced with AKI with a hemodynamic basis, such as hepatorenal syndrome. In addition, the guidelines still include generalized recommendations regarding withdrawal of diuretics and plasma volume expansion with albumin for 48 h, which may be ineffective and counterproductive and may have iatrogenic effects, such as fluid overload and acute cardiogenic pulmonary edema. For this reason, the use of new tools, such as hemodynamic point-of-care ultrasound (PoCUS), allows us to phenotype volume status more accurately and ultimately guide medical treatment in a noninvasive, rapid and individualized manner.

## INTRODUCTION

Acute kidney injury (AKI) is frequently observed in patients with hepatic cirrhosis, with an incidence ranging from 20% to 50% among hospitalized patients according to different series. It is associated with high morbidity and mortality rates, especially in patients with acute-on-chronic liver failure (ACLF), and increased incidence of chronic kidney disease (CKD) [1–3].

The hemodynamic changes found in cases of cirrhosis with portal hypertension (PH) lead to the development of a specific form of AKI and CKD—hepatorenal syndrome (HRS). HRS is associated with a negative prognosis, with a median survival of less than 3 months from onset [4, 5]. Despite the continuous evolution of the diagnostic criteria proposed by the International Club of Ascites (ICA), its diagnosis is one of exclusion (see Table 1) and requires, among other criteria, no improvement in renal function after empirical volume expansion with albumin for at least 48 h [6, 7].

**Table 1:** Diagnostic criteria for hepatorenal syndrome.

	ICA 1996 [15]	ICA 2007 [16]	ICA 2015 [17]
Major criteria	Chronic-to-acute hepatic disease with hepatic failure and PH	Chronic liver disease and ascites	Chronic liver disease and ascites
	Serum creatinine >1.5 mg/dL or creatinine clearance <40 mL/min	Serum creatinine >1.5 mg/dL	Stage 2 by AKIN classification (2-fold increase in serum creatinine)
	Two-fold increase in serum creatinine ≥2.5 mg/dL in <2 weeks	Two-fold increase in serum creatinine ≥2.5 mg/dL in <2 weeks	
	No renal function improvement after diuretic withdrawal and volume expansion with 1.5 L isotonic saline solution	No renal function improvement after suspension of diuretics and volume expansion for 48 h with albumin (1 g/kg/day)	No renal function improvement after suspension of diuretics and volume expansion for 48 h with albumin (1 g/kg/day)
	No exposure to nephrotoxic drugs	No exposure to nephrotoxic drugs	No exposure to nephrotoxic drugs
	No shock or bacterial infection	No shock	No shock
		Urinary sediment with <50 red blood cells/field	Urinary sediment with <50 red blood cells/field
	Proteinuria <500 mg/day	Proteinuria <500 mg/day	Proteinuria <500 mg/day
	Normal kidney ultrasound	Normal kidney ultrasound	Normal kidney ultrasound
Minor criteria	Urinary sodium <10 mEq/L		
	Urinary volume <500 mL		
	Urinary osmolality > plasma osmolality		
	Urinary sediment with <50 red blood cells/field		
	Plasma sodium <130 mEq/L		
Other			Omission of HRS type 1; introduction of HRS-AKI and HRS-NAKI

^a^Adapted from Velez *et al.* [6].

AKIN: Acute Kidney Injury Network; HRS-NAKI: hepatorenal syndrome non-AKI.

This diagnostic approach assumes a generalized “responsive to volume expansion” profile which may be associated with iatrogenesis. Conversely, the hemodynamic changes inherent to HRS are predisposing factors to AKI, and often overlap with other causes of AKI (use of diuretics, gastrointestinal losses, gastrointestinal bleeding, paracentesis for evacuation, exposure to nonsteroidal anti-inflammatory drugs, beta-blockers, antibiotics and iodinated contrast agents, cholestasis-associated nephropathy, and/or other nephropathies).

Furthermore, despite a thorough case history, physical examination and the use of various biomarkers in the assessment of AKI, recent studies have demonstrated that the assessment of volume status in patients with cirrhosis is heterogeneous. Consequently, other noninvasive tools are required as complementary methods, such as point-of-care ultrasound (PoCUS), which allows for a multiorgan clinical approach in order to simplify diagnosis while avoiding counterproductive treatment [8–10].

## PATHOPHYSIOLOGY OF AKI IN PATIENTS WITH CIRRHOSIS

**Figure 1:**
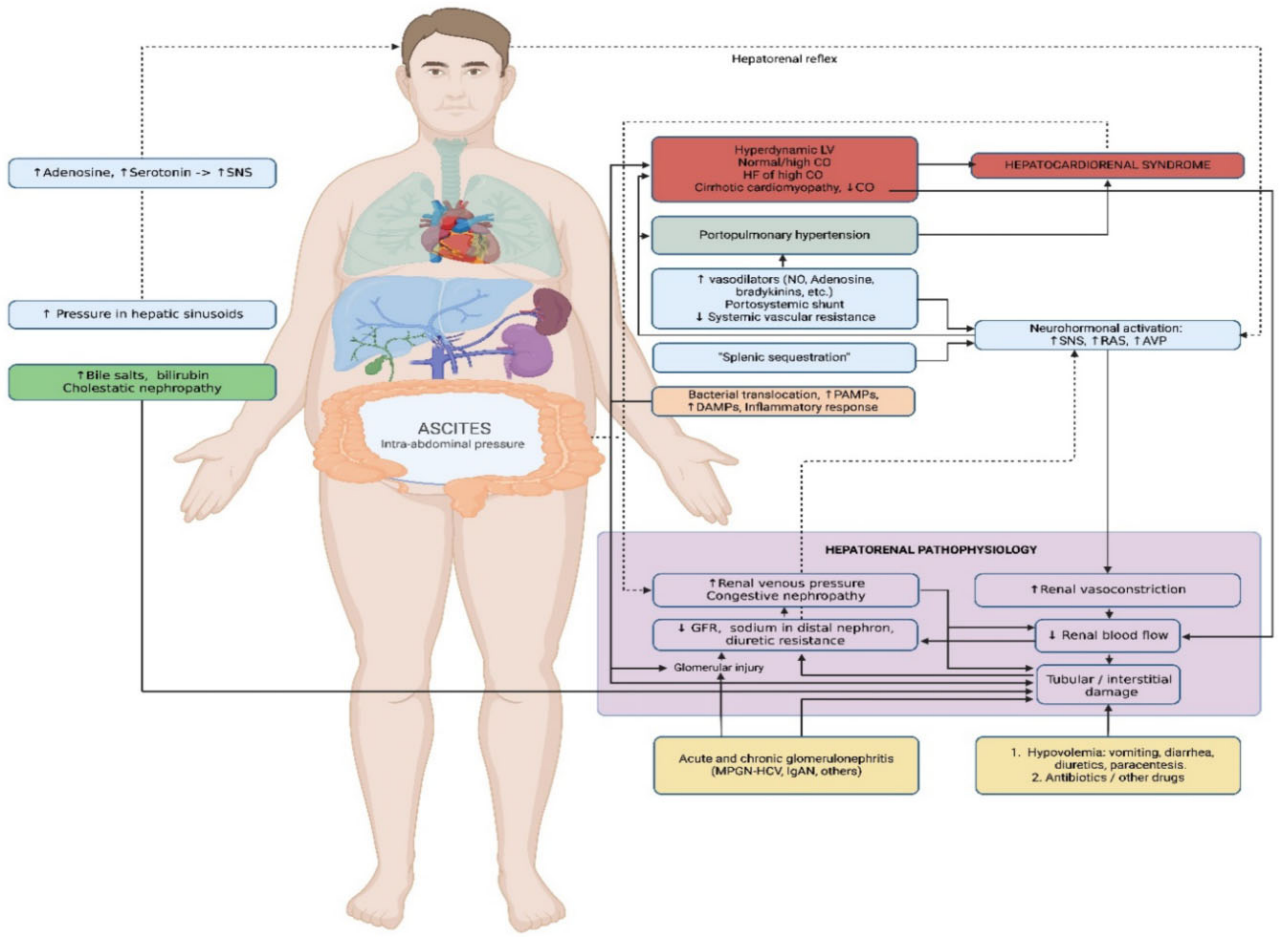
Pathophysiology of AKI in patients with cirrhosis. Hepatocardiorenal pathophysiology. HF: heart failure; CO: cardiac output; NO: nitric oxide; ECV: effective circulating volume; AVP: vasopressin; PAMPs: pathogen-associated molecular patterns; DAMPs: damage-associated molecular patterns; IgAN: immunoglobulin A nephropathy; MPGN-HCV: membranoproliferative glomerulonephritis associated with hepatitis C virus. Created with BioRender.com.

The pathogenic mechanisms that may contribute to the development of AKI in cirrhotic patients are multiple and often overlap (see Fig. 1). However, in this paper we will focus on hemodynamic changes given that PoCUS is especially useful in their diagnosis.

Structural damage of the liver parenchyma increases vascular resistance in its sinusoids and favors the onset of PH, thus inducing local endothelial dysfunction and increased production of vasodilator substances (mainly nitric oxide), which translates into splanchnic sequestration. As cirrhosis progresses, the patient experiences a proinflammatory state enhanced by bacterial translocation from the gastrointestinal tract and the release of endotoxins. This, together with the generation of portosystemic shunts, leads to generalized endothelial dysfunction, decreased peripheral resistance and direct lesion of the renal tubule.

The aforementioned events result in the activation of initially adaptive mechanisms [sympathetic nervous system (SNS), renin–angiotensin–aldosterone system (RAAS), antidiuretic hormone and vasopressin], which cause hyperdynamic circulation by increasing stroke volume, heart rate and volume expansion, at the expense of afferent arteriolar vasoconstriction and decreased renal blood flow. Experimental studies have shown SNS activation, regardless of the decrease in effective circulating volume, through the liver–brain–kidney pathway—resulting from the hepatic release of adenosine and serotonin in the context of PH—and have coined the term “hepatorenal reflex” to refer to this pathway [11, 12].

Persistent neurohumoral activation leads to increased ventricular and pulmonary filling pressure, with possible occurrence of portopulmonary hypertension, venous congestion, refractory ascites, intra-abdominal hypertension, abdominal compartment syndrome and congestive nephropathy, ultimately reflecting circulatory “maladaptation.”

In the final phase, decreased afterload due to low peripheral resistance may be accompanied by decreased cardiac output due to chronotropic and inotropic dysfunction. This scenario, known as cirrhotic cardiomyopathy, may worsen renal hypoperfusion, PH, and consequent sodium and water retention.

Multiple pathways associated with the development of AKI in patients with cirrhosis (neurohumoral activation, systemic inflammation and endothelial dysfunction) are also observed in cardiorenal syndrome, which is why the concept of hepatocardiorenal syndrome has been recently suggested [13, 14].

## WHY IS IT CONVENIENT TO USE PoCUS IN PATIENTS WITH CIRRHOSIS WHO HAVE AKI?

In clinical practice and using traditional tools, it is often challenging to distinguish whether hemodynamic AKI in cirrhosis is caused by volume depletion or the “maladaptation” changes mentioned above. Table 2 summarizes the potential clinical scenarios that favor volume depletion or overload in cirrhosis.

**Table 2:** Main clinical scenarios in which patients may present volume overload or depletion.

Volume depletion	Volume overload
Gastrointestinal bleeding	High dietary sodium intake
Excessive diuretic use	Heart failure
Paracentesis for evacuation	Hepatocardiorenal syndrome
Vomiting	Oliguric AKI
Diarrhea ^a^Prior lactulose use to treat hepatic encephalopathy	Intense reanimation with fluid therapy

**Figure 2:**
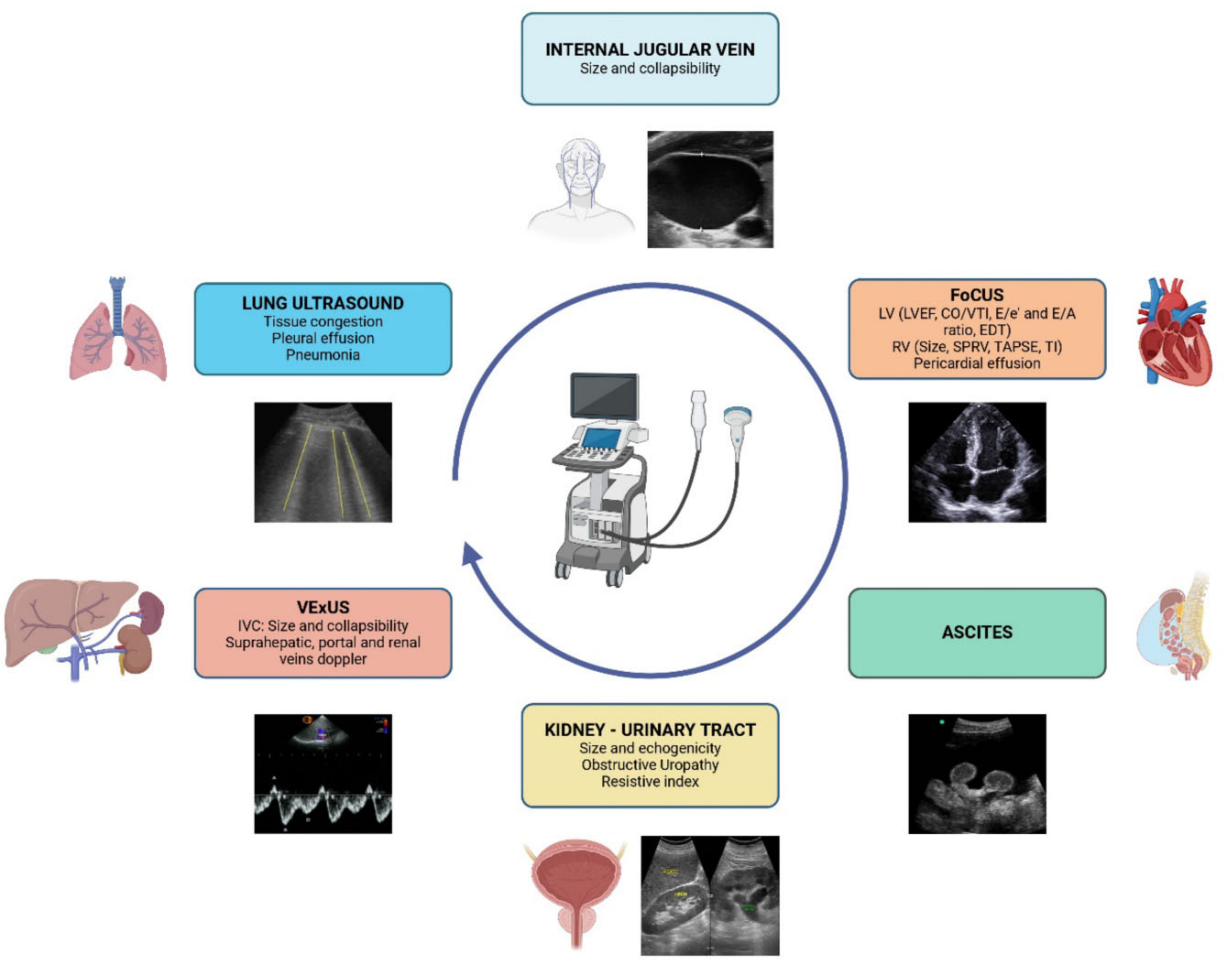
AKI and cirrhosis: multiorgan ultrasound assessment. LVEF: left ventricular ejection fraction; CO: cardiac output; EDT: E wave deceleration time; RV: right ventricle; SPRV: systolic pressure of the RV; TAPSE: tricuspid annular plane systolic excursion; TI: tricuspid insufficiency. Created with BioRender.com.

Therefore, a multiparametric approach to patients’ hemodynamic status should be proposed by adding PoCUS to our algorithm (see Fig. 2). This way, volume expansion would not be performed unlimitedly, and factors such as the use of diuretics, amines and/or paracentesis for evacuations can be considered from the start, depending on the scenario.

The use of PoCUS for the assessment of hemodynamic status entails three strategies: lung ultrasound (LUS), which allows for a rapid and accurate assessment of pulmonary tissue congestion; the venous excess ultrasound grading system (VExUS), which assesses and grades systemic vascular congestion; and the analysis of cardiac morphology and function by means of echocardiography or focused cardiac ultrasound (FoCUS) [15].

**Figure 3:**
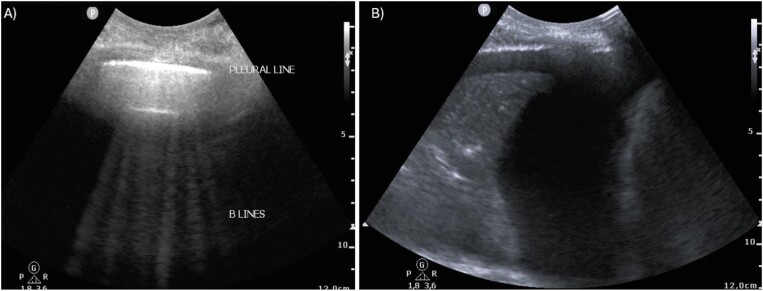
Congestion assessment using LUS. A 60-year-old male patient with cirrhosis was admitted to the intensive care unit (ICU) for hypovolemic shock secondary to gastrointestinal bleeding. On the fourth day of admission, he presented with dyspnea and persistent AKI. A LUS was performed. More than three B-lines per area were observed bilaterally (A). Note the large right pleural effusion. Based on the remaining physical examination and diagnostic tests, a diagnosis of cardiogenic pulmonary edema following intensive fluid resuscitation was made. Fluid therapy was discontinued, and diuretic treatment was started with improvement in symptoms and kidney function.

Congestion is initially assessed using LUS, where B-lines or pleural comets are observed. B-lines are vertical hyperechoic artifacts of the pleura, which translate into interstitial changes secondary to transudate or exudate (see Fig. 3). No lung scanning technique (4 zones/6 zones/8 zones/28 zones) has been standardized in patients with cirrhosis. In the authors’ experience, the use of an abbreviated 8-zone protocol is less cumbersome and has demonstrated similar diagnostic accuracy in the assessment of pulmonary congestion compared with full protocols in other settings such as hemodialysis [16]. The presence of three or more B-lines in two or more zones is associated with parenchymal tissue congestion. LUS is more sensitive for pulmonary edema identification as opposed to physical examination, as demonstrated in a cohort of 926 patients hospitalized in a critical care unit, where breath sounds were normal in 51% of patients with pulmonary edema criteria by ultrasound [17]. The presence of bilateral B-lines correlates with increased left ventricular filling pressure determined both by ultrasound and invasive methods, such as pulmonary capillary wedge pressure, regardless of left ventricular ejection fraction [18].

Assessment of venous congestion is generally performed by assessing the size and collapsibility index of the inferior vena cava (IVC). However, IVC insonation may be misleading in patients with cirrhosis due to hepatic fibrosis and intra-abdominal hypertension due to ascites, and cannot be performed in 20% of patients [19–21]. A recent observational study conducted with 44 patients evidenced that the internal jugular vein (IJV) collapsibility index ≤24.8%, determined at the 30° body position, showed the best performance in predicting a central venous pressure ≥8 mmHg, with a sensitivity of 100% and a specificity of 97.1% [22].

In addition, the recently described VExUS protocol allows for the identification and stratification of systemic venous congestion by complementing IVC assessment with pulsed Doppler of the suprahepatic veins, portal vein and interlobar renal veins to assess for altered flow patterns observed in venous congestion. Prior reviews summarized the pathophysiological basis of this ultrasound protocol, the findings in the pulsed Doppler scan, its correct interpretation and pitfalls [23–26].

**Figure 4:**
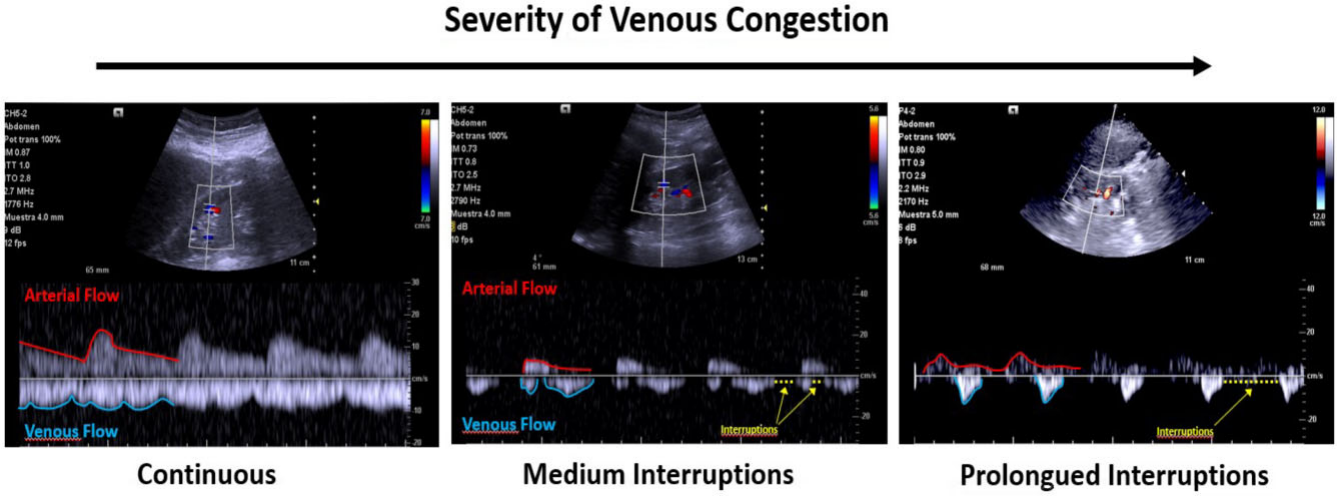
Assessment of renal venous flow in the presence of congestion. Under normal conditions, intrarenal venous flow is continuous and can be observed by pulsed Doppler below baseline (blue line). Renal venous flow is interrupted (yellow dots) and becomes biphasic or monophasic as systemic congestion increases. Note that low resistance arterial flow appears above baseline (red line).

In patients with cirrhosis, altered liver anatomy precludes interpretation of supra-hepatic and portal vein Doppler. However, it is our experience that intra-renal venous Doppler (IRVD) is a useful tool to quantify and monitor the severity of venous congestion and congestive nephropathy in patients with hepatocardiorenal syndrome. IRVD is performed by scanning the small veins of the kidneys (interlobar or arcuate) and assessing the pattern of flow [27]. In the absence of congestion, venous flow is continuous, and it becomes progressively more interrupted with worsening venous congestion [28] (Fig. 4).

**Figure 5:**
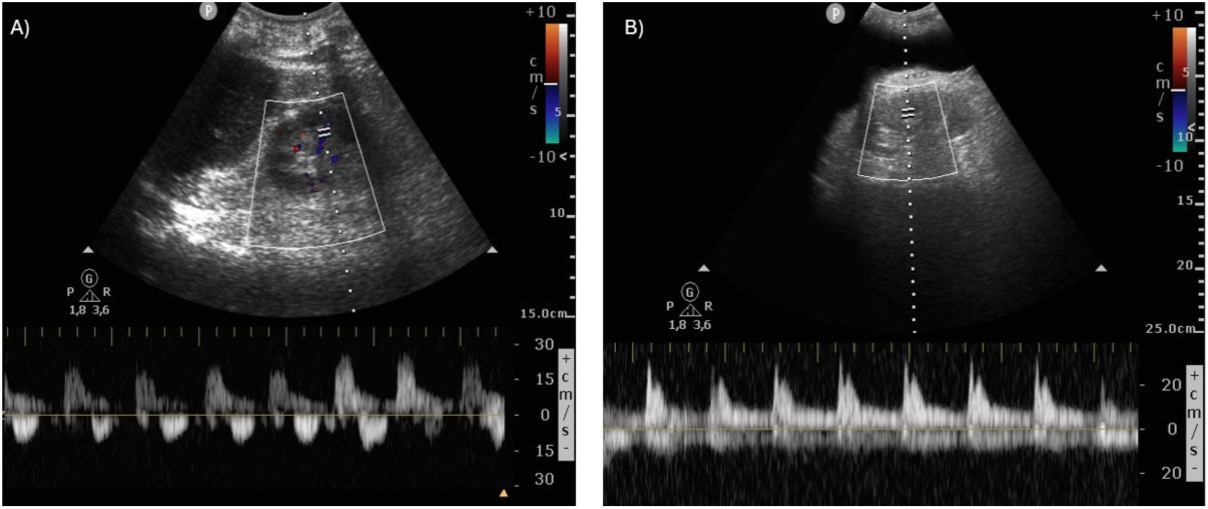
Renal venous flow assessment using VExUS. A 45-year-old male patient was admitted to the hospital with decompensated cirrhosis and AKI. He was referred to nephrology after no improvement in renal function for at least 48 h after empirical volume expansion with albumin. VExUS was performed demonstrating a monophasic renal flow. A diagnosis of congestive nephropathy was made (**A**). After decongestive treatment with intravenous diuretics and a negative fluid balance of –9 L on the seventh day, with an improvement in renal function, the VExUS was repeated. Continuous renal venous flow was observed (**B**).

Based on the latter, we believe that, in the case of patients with cirrhosis, venous congestion should be assessed using alternative sites, such as IJV ultrasound and IRVD. Figure 5 provides a case vignette of a patient with hepatocardiorenal syndrome and venous congestion.

Heart failure is characterized by the inability to generate adequate stroke volume or reaching such volume at the expense of increased filling pressures, resulting in venous congestion, which is why correlating venous congestion with cardiac assessment is critical. The FoCUS technique allows for a morphological and functional assessment of both the right and left ventricles, as well as rapid assessment of the presence of pericardial effusion, tamponade and valvular abnormalities. Furthermore, when combining LUS and VExUS, which is essential when assessing congestion, hypovolemic patients cannot be differentiated from those with a redistributive phenotype unless FoCUS is used [10]. Cardiac output and stroke volume (SV) can be routinely assessed by pulsed-wave Doppler echocardiography. SV is usually calculated from the product of the left ventricular outflow tract (LVOT) cross-sectional area and the velocity-time integral (VTI). Finally, cardiac output is usually obtained by multiplying SV by heart rate.

**Figure 6:**
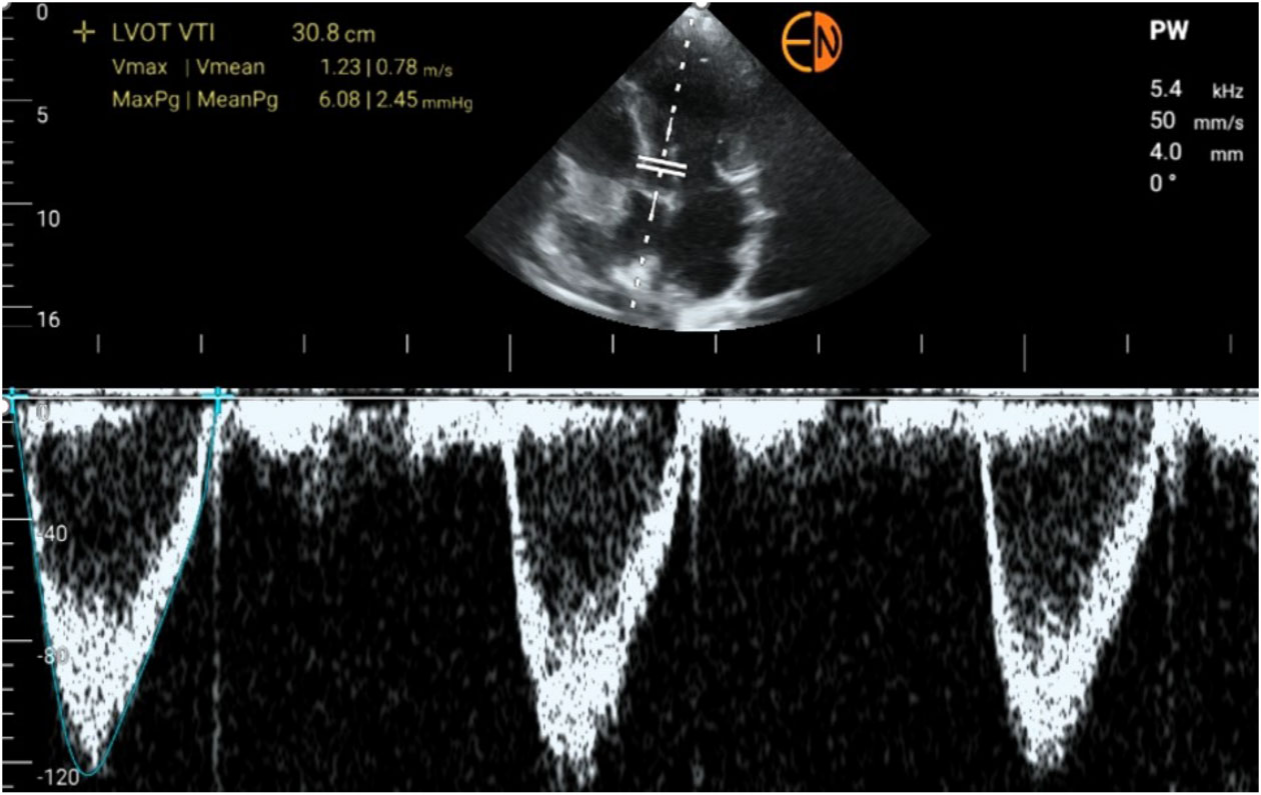
Morphological and functional assessment of the heart using the FoCUS technique. Apical 5-chamber pulsed Doppler view of the LVOT. Note the VTI measurement of 30.8 cm (normal range 17–22 cm). This finding in the setting of acute kidney injury in a patient with cirrhosis can reasonably rule out true hypovolaemia.

These two basic hemodynamic parameters can be useful in demonstrating high or low cardiac output states in patients with cirrhosis, helping the clinician to differentiate between true hypovolemia and a redistributive phenotype such as HRS [29–32] (see Fig. 6).

Due to the above, different studies of the usefulness of PoCUS in the assessment of AKI in cirrhotic patients have been published in the last years, demonstrating a very heterogeneous scenario from the hemodynamic point of view, as well as a poor correlation with the use of traditional diagnostic methods, such as physical examination and analytical determinations [9, 10, 33]. Up to 60%–65% of patients are incorrectly diagnosed with HRS [34]. A recent study reported that 62% of patients diagnosed with HRS based on clinical criteria had high cardiac filling pressure determined by right heart catheterization, thus improving serum creatinine when volume expansion was discontinued and diuretic treatment was initiated [35].

## HEPATORENAL SYNDROME: EVOLUTION OF DIAGNOSTIC CRITERIA AND LIMITATIONS

Since its first definition, proposed in 1996 by the ICA, HRS is still a diagnosis of exclusion, which entails its own clinical practice challenges. Table 1 shows the diagnostic criteria proposed by the ICA over the years [36–38].

The main diagnostic limitations will be discussed below:

(i) Changes in renal function. Until 2015, the definition of HRS used absolute serum creatinine values as a cutoff point instead of relative changes. This was modified in the last revision, and the Kidney Disease: Improving Global Outcomes criteria were included to define AKI. However, relative changes in serum creatinine levels depend on their absolute values, and their interpretation in cases of cirrhosis is limited due to high prevalence of sarcopenia [39], interference of bilirubin in the colorimetric creatinine assay [40] and/or increased tubular secretion [41]. In this context, estimation of glomerular filtration rate (GFR) using cystatin C seems to have a better correlation with inulin clearance than estimations using serum creatinine [42].
(ii) Urinalysis (urinary sediment and sodium). The latest diagnostic criteria exclude HRS in the presence of microscopic hematuria (defined as >50 red blood cells/field) and/or proteinuria >500 mg. However, the hemodynamic changes typical of HRS may overlap with kidney injury, regardless of its cause. This approach does not take into account basal urinary tests, sets arbitrary cutoff points for proteinuria and microscopic hematuria, does not assess the presence of dysmorphic red blood cells and does not take into account artifacts secondary to the presence of bacterial flora, bladder catheterization and/or oligoanuria. Leukocyturia, a frequent finding in conditions, such as glomerulonephritis (GN) and interstitial nephritis (IN), is not mentioned either. In addition, this approach does not analyze the presence of urinary casts in sediment, a finding that may guide diagnosis, granular casts suggest acute tubular necrosis (ATN), leukocyte and hematic casts suggest GN and/or IN, and bilirubin casts may indicate cholestatic nephropathy [43]. However, it should be highlighted that bile casts can be found in cases without AKI and that they may reflect a decreased GFR and tubular stasis typical of HRS in patients with cirrhosis without necessarily entailing tubular damage.
  Although the ICA eliminated the use of urine biochemistry as a criterion for HRS in 2007, the cutoff points for urinary fractional excretion of sodium (FENa) <1% and low urinary sodium (<20 mEq/L), widely used in the general population as a surrogate marker of prerenal AKI, are wrongly extrapolated to patients with cirrhosis having PH. This is because neurohumoral activation entails a baseline FENa <1% and urinary sodium <20 mEq/L, thus reflecting the high avidity of proximal tubule avidity for sodium. Likewise, just as several studies have shown that a FENa >2% or urinary sodium >40 mEq/L are highly inconsistent with HRS, FENa values <0.1% suggest functional AKI [44, 45]. This highlights the need for laboratories to show urinary sodium values in absolute terms, especially when <20 mEq/L is found. The use of specific renal biomarkers that allow for the differentiation between structural damage or functional damage, would be very useful. Urinary neutrophil gelatinase-associated lipocalin (NGAL), a marker of tubular injury, has been shown to be able to differentiate between HRS and ATN. However, its use in clinical practice is not standardized due to the lack of validated cutoff points and low availability in healthcare centers [45, 46].

(iii) Nonexposure to nephrotoxic drugs. Patients with cirrhosis are especially sensitive to nephrotoxic drugs, such as antibiotics and nonsteroidal anti-inflammatory drugs. However, toxic ATN may develop with FENa levels <1% and urinary sodium levels <20 mEq/L, just as acute interstitial nephritis may be accompanied by poorly expressive element and sediment urine test values. Therefore, since there are no sufficiently robust noninvasive tools for the screening of these conditions, it may be necessary to resort to renal biopsy.
(iv) No renal structural changes observed by imaging. If the hemodynamic changes of HRS can overlap with CKD, imaging studies may show morphological changes typical of this condition (hyperechogenic parenchyma, cortical thinning, renal atrophy, etc.). We may also incidentally find injuries, such as simple cysts or neoplasms, concurrently with the hemodynamic disorders typical of hepatorenal pathophysiology. However, imaging studies should be conducted in the face of AKI to rule out conditions such as obstructive uropathy.
(v) No improvement of renal function after withdrawal of diuretics and volume expansion for 48 h with albumin (1 g/kg/day). Despite the limitations of both physical examination and laboratory tests in the assessment of patients’ hemodynamic status, most clinical practice guidelines still recommend this therapeutic approach as an initial approach. Sometimes, a patient may present with severe hypovolemia and require volume expansion for more than 48 h to improve or, on the contrary, a patient may not have obvious symptoms or physical signs of fluid volume overload but have elevated biomarkers and ultrasound signs of intravascular or tissue congestion, delaying diagnosis and worsening hemodynamic instability. The early versus standard initiation of terlipressin for HRS-AKI in with ACLF patients study, a randomized controlled trial, evidenced how early initiation of terlipressin in patients with ACLF and AKI, compared with standard initiation at 48 h and no improvement in renal function despite 12 h of volume expansion with albumin, achieved higher rates of complete recovery of renal function at 7 days (68.6% vs 40%; *P* = .03), lower 28-day mortality rates (40% vs 65.7%; *P* = .031) and a significant reduction in ACLF stage [47]. Contrarily, in the randomized ATTIRE study, the prophylactic use of albumin in patients with decompensated cirrhosis could not prevent infections, AKI and death in the short term, thus increasing the number of patients with congestion [48]. This highlights the fact that the same treatment cannot be offered to all patients with cirrhosis and AKI, and that noninvasive tools should be used to achieve a rapid and accurate classification of hemodynamic status.

## HEMODYNAMIC PHENOTYPES IN CIRRHOSIS, DIAGNOSIS AND TREATMENT

### Responsive to volume expansion phenotype (true hypovolemia): responsive to crystalloids and/or albumin

AKI secondary to hypovolemia is frequent in cirrhosis and is usually caused by decreased food intake in the context of hepatic encephalopathy, laxative abuse, diuretic overtreatment and/or gastrointestinal hemorrhage, especially if the patient concomitantly takes neurohumoral blocking agents.

In the presence of hypovolemia, a pattern of A-lines (horizontal artifacts parallel to the pleural line) is usually identified by LUS, as well as a nondilated IVC <20 mm, with a normal variability >50%, and a decrease in stroke volume measured by determining the size of the LVOT and the VTI estimated at that point.

Treatment will focus on potential cause correction, temporary withdrawal of RAAS blockade and diuretics, and volume expansion with periodic re-evaluation. The implementation of PoCUS during volume expansion should also be considered as a strategy to achieve optimal volume expansion and avoid hypervolemia.

### Nonresponsive to volume expansion phenotype: nonresponsive to volume expansion with crystalloids

This is possibly the most frequent phenotype managed by nephrologists, a patient with cirrhosis presents AKI due to lack of response to diuretic withdrawal and empirical volume expansion.

#### Hepatorenal syndrome (redistributive phenotype)

An A-line pattern is observed by LUS, as well as nondilated IVC <20 mm, with normal variability >50%, hyperdynamic left ventricle (LV) with normal or increased cardiac output, and ascites. Treatment will focus on potential cause correction, temporary withdrawal of RAAS blockade and diuretics, paracentesis for evacuation in case of intra-abdominal hypertension or tension ascites, and early initiation of terlipressin + albumin [49]. Although concomitant albumin administration was recommended or required in almost all clinical trials evaluating the efficacy of terlipressin therapy, this is based on an isolated clinical trial, with a small sample size and heterogeneous populations (patients meeting the classic definitions of HRS type 1 and 2), and HRS reverted when albumin was combined with vasopressors and not when vasopressors were administered as monotherapy. Current ICA recommendations for intravenous albumin dosing are 1 g/kg/day (100 g maximum) for at least 24–48 h, followed by 25 g/day thereafter.

#### Abdominal compartment syndrome

An A-line pattern is identified by LUS, as well as a nondilated IVC <20 mm, with generally decreased collapsibility <50%, ascites, and possible alteration in the renal vein pulsed Doppler scan resulting from the compression exerted by the ascites on the renal parenchyma (biphasic or monophasic venous flow). After performing paracentesis for evacuation, the IVC should be insonated again because the VExUS protocol will be needed if it reaches a size >20 mm, since intra-abdominal hypertension could be masking the presence of venous congestion [50–52].

#### Hepatocardiorenal syndrome

The presence of B-lines, generally bilateral and symmetrical, together with increased LV filling pressure, confirms the presence of left heart failure (the presence of B-lines, generally with asymmetrical distribution and in the presence of normal LV filling pressures, may suggest other conditions, such as respiratory infections). Conversely, the presence of a dilated IVC >20 mm with low collapsibility <50% indicates increased right atrial pressure; in that case, the right ventricle should be assessed, and the systemic venous congestion should be graded by means of VExUS (prioritizing the intrarenal Doppler scan). In this scenario, decreased cardiac output requires ruling out the presence of cirrhotic cardiomyopathy. Treatment will focus on decongestion (diuretics), paracentesis for evacuation in the case of refractory tense ascites, ionotropics if needed and specific treatment of possible causes.

**Figure 7:**
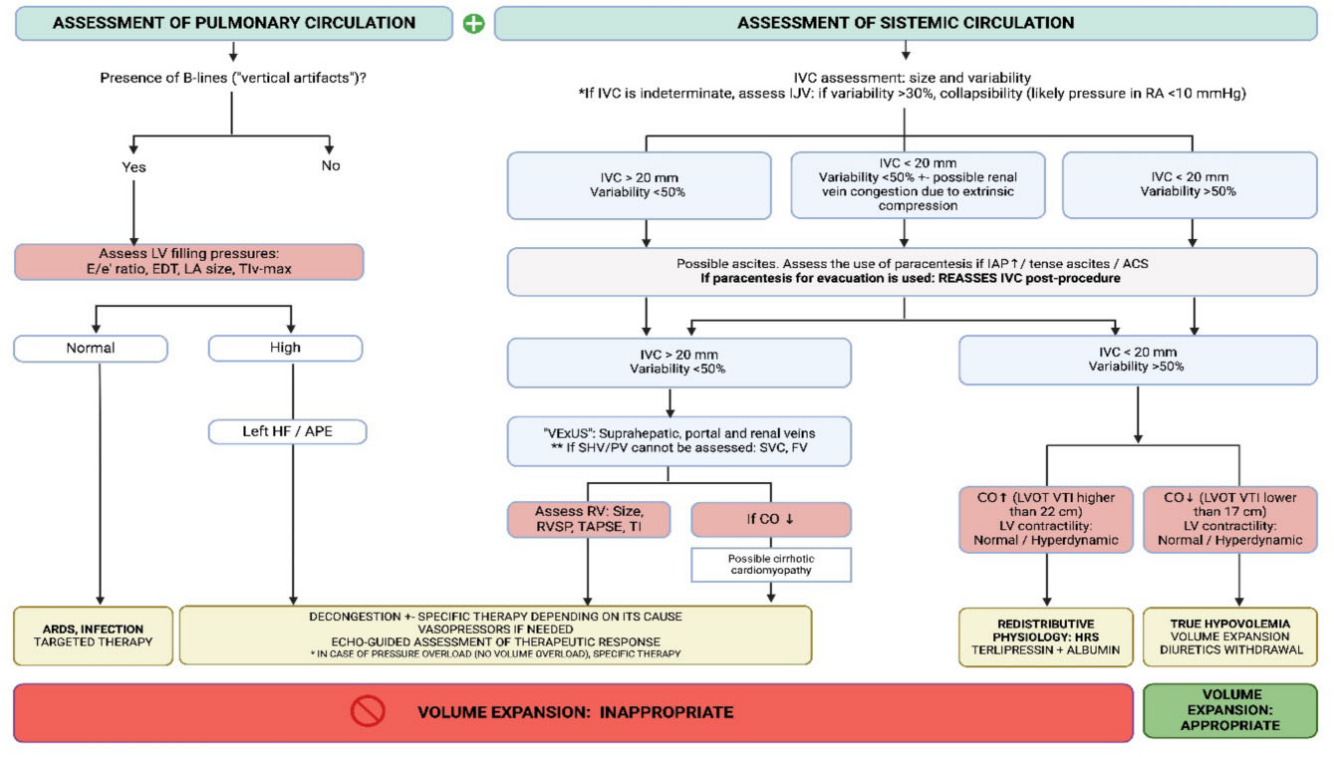
Diagnostic and therapeutic algorithm of hemodynamic AKI in patients with cirrhosis using PoCUS. RA: right auricle; LA: left auricle; APE: acute pulmonary edema; CO: cardiac output; HF: heart failure; TI: tricuspid insufficiency; TIv-max: maximum velocity of TI jet; LVOT-VTI: left ventricular outflow tract velocity time integral (normal 17–22 cm). If the insonation angle is incorrect or the images obtained are of poor quality, the Doppler VTI measurement may be subject to error. RAP: right auricle pressure; IAP: intra-abdominal pressure; RVSP: right ventricle systolic pressure; ACS: abdominal compartmental syndrome; ARDS: acute respiratory distress syndrome; TAPSE: tricuspid annular plane systolic excursion; EDT: E wave deceleration time; IVC: inferior vena cava; SVC: superior vena cava; RV: right ventricle; FV: femoral vein; SHV: suprahepatic veins. Created with BioRender.com.

Below, we propose a diagnostic and therapeutic algorithm for AKI in patients with cirrhosis with hemodynamic basis using PoCUS, which includes both pulmonary or minor circulation assessment and systemic or major circulation assessment (see Fig. 7).

## CONCLUSIONS

The diagnosis of HRS continues to be one of exclusion and requires, among other criteria, the absence of improvement in renal function after empirical volume expansion with albumin for at least 48 h. In this context, whether to administer intravenous fluids or use diuretics (“to fill or to drain”) is a common clinical dilemma faced by clinicians providing care for patients with cirrhosis having AKI. Addressing the causes of AKI by combining traditional tools with point-of-care ultrasound scan (PoCUS) allows for a rapid and accurate diagnostic approach, applying individualized therapeutic strategies instead of the universal withdrawal of diuretics and volume expansion with albumin proposed by the ICA guidelines. Based on ultrasound findings, an algorithm is proposed to characterize hemodynamic status in cirrhosis with AKI that suggests not considering hepatorenal syndrome as an isolated pathophysiological condition with a diagnosis of exclusion.

## Data Availability

No new data were generated or analysed in support of this research.
